# Deep Bottleneck Features for Spoken Language Identification

**DOI:** 10.1371/journal.pone.0100795

**Published:** 2014-07-01

**Authors:** Bing Jiang, Yan Song, Si Wei, Jun-Hua Liu, Ian Vince McLoughlin, Li-Rong Dai

**Affiliations:** 1 National Engineering Laboratory of Speech and Language Information Processing, University of Science and Technology of China, Hefei, AnHui, China; 2 iFlytek Research, Anhui USTC iFlytek Co., Ltd., Hefei, AnHui, China; University of Texas Health Science Center at San Antonio, Research Imaging Institute, United States of America

## Abstract

A key problem in spoken language identification (LID) is to design effective representations which are specific to language information. For example, in recent years, representations based on both phonotactic and acoustic features have proven their effectiveness for LID. Although advances in machine learning have led to significant improvements, LID performance is still lacking, especially for short duration speech utterances. With the hypothesis that language information is weak and represented only latently in speech, and is largely dependent on the statistical properties of the speech content, existing representations may be insufficient. Furthermore they may be susceptible to the variations caused by different speakers, specific content of the speech segments, and background noise. To address this, we propose using Deep Bottleneck Features (DBF) for spoken LID, motivated by the success of Deep Neural Networks (DNN) in speech recognition. We show that DBFs can form a low-dimensional compact representation of the original inputs with a powerful descriptive and discriminative capability. To evaluate the effectiveness of this, we design two acoustic models, termed DBF-TV and parallel DBF-TV (PDBF-TV), using a DBF based i-vector representation for each speech utterance. Results on NIST language recognition evaluation 2009 (LRE09) show significant improvements over state-of-the-art systems. By fusing the output of phonotactic and acoustic approaches, we achieve an EER of 1.08%, 1.89% and 7.01% for 30 s, 10 s and 3 s test utterances respectively. Furthermore, various DBF configurations have been extensively evaluated, and an optimal system proposed.

## Introduction

Language identification (LID) is the task of determining the identity of the spoken language present within a speech utterance. LID is a key pre-processing technique for future multi-lingual speech processing systems, such as audio and video information retrieval, automatic machine translation, diarization, multi-lingual speech recognition, intelligent surveillance and so on.

A major problem in LID is how to design a language specific and effective representation for speech utterances. It is challenging due to large variations introduced by different speech content, speakers, channels and background noises. Over the past few decades, intensive research efforts have studied the effectiveness of different representations from various research domains, such as phonotactic and acoustic information [1]–[3], lexical knowledge [4], prosodic information [5], articulatory parameters [6], and universal attributes [7]. Among existing representations, Eady [5], Matrouf et. al. [4] and Kirchoff et. al. [6] show that appropriate incorporation of extra language-related cues may help to improve the effectiveness of representation. In this paper, we mainly focus on the phonotactic and acoustic representations, which are considered to be the most common ones for LID [8], [9].

Phonotactic representations focus on capturing the statistics of phonemic constraints and patterns for each language. It is known that the phonotactic representation of a given utterance is the token sequence or lattice output from a phone recognizer (PR). The corresponding approaches, such as Parallel Phone Recognizers followed by Language Models (PPR-LM) [3] and Parallel Phone Recognizers followed by Support Vector Machines (PPR-SVM) [10], [11] have achieved the state-of-the-art performance. However, the effectiveness of such representations relies heavily on the performance of the phone recognizer (PR) [12]. When the labelled dataset size is limited, it is difficult to achieve good PR results. Furthermore, the recognizing stage is time consuming, which constrains the wide applicability of the phonotactic approaches.

By contrast, acoustic representations mainly capture the spectral feature distribution for each language, which is more efficient and does not require prior linguistic knowledge. Two important factors for effective acoustic representation are, (1) a front-end feature extractor which forms the frame level representation based on spectral features, and (2) a back-end model which constructs the acoustic representation for spoken LID. A popular feature is Shift Delta Cepstra (SDC), which is effectively an extension of traditional MFCC or PLP features [13]–[15]. Typical back-end models include Gaussian Mixture Model-Universal Background Model (GMM-UBM) [15] and Gaussian Mixture Model-Support Vector Machine (GMM-SVM) [16], [17]. With the help of modern machine learning techniques, such as discriminative training [18]–[20], Factor Analysis (FA) [21]–[23] and Total Variability (TV) modeling [24], [25], the performance of acoustic approaches tends to be comparable to or even exceed that of phonotactic ones. In fact, even greater performance improvement can be achieved by exploiting both phonotactic and acoustic approaches, through fusing their results [26]–[28].

Despite significant recent advances in LID techniques, performance is still far from satisfactory, especially for short duration utterances [9]. This may be because language characteristics are a kind of weak information latently contained in the speech signal and largely dependent on its statistical properties. For short duration utterances especially, existing representations are deficient by being overly susceptible to variations caused by different speakers, channels, speech content and background noises. To address this, more powerful features, having higher discriminative and descriptive capabilities, are preferred.

Recently, deep learning techniques have achieved significant performance gains in a number of applications, including large scale speech recognition and image classification [29], [30], largely due to their powerful modeling capabilities, aided by the availability of the large scale datasets. In this paper, we aim to apply deep learning techniques to the spoken LID task. Our preliminary work demonstrated that an acoustic system based on deep bottleneck features (DBF) can effectively mine the contextual information embedded in speech frames [31]. Specially, DBFs were generated by a structured Deep Neural Network (DNN) containing a narrow internal bottleneck layer. Since the number of hidden nodes in the bottleneck layer is much smaller than those in other layers, DNN training forces the activation signals in the bottleneck layer to form a low-dimensional compact representation of the original inputs. It should be noted that this is unlike work by Diez et. al. [32], [33], in which the log-likelihood ratios of posterior probabilities, called Phone Log-Likelihood Ratios (PLLR), output from the multi-layered perceptron(MLP), were used as frame level features for LID. We will present a more detailed discussion and comparison later in this article.

This paper extends our preliminary work in five main ways:

- The DBF extractor and DNN structure are analyzed and evaluated together with the crucial DBF training and extraction process (including assessing two alternative training corpuses and their configurations). In addition, the relationship to the conventional SDC [13]–[15] and recently proposed PLLR [32], [33] approaches are explored;
- Two new acoustical systems are presented, i.e. DBF-TV and parallel DBF-TV (PDBF-TV), and systematically evaluated across various configurations of DBF extractor. The systems are evaluated for a range of input feature temporal window sizes, and number of bottleneck layer hidden nodes;
- The relationship is explored between DBF and different test conditions, based on analysis of evaluation results;
- An optimal LID system configuration is proposed based on the NIST language recognition evaluation 2009 (LRE09) dataset, and compared to other high performance published approaches;
- A phonotactic representation is constructed, using a GMM-HMM based phone recognizer (PR) trained with DBF. The output is fused with that of the acoustic representation (using two alternative fusion methods) to achieve extremely good performance.

Experimental results will demonstrate that an acoustic representation based on DBF significantly improves on state-of-the-art performance, especially for short duration utterances. The proposed phonotactic and acoustic fusion achieves equal error rate (EER) figures of 1.08%, 1.89% and 7.01% for 30 s, 10 s and 3 s test utterances respectively. This clearly exceeds the performance of the best currently reported LID system [9], as well as our own previous work [31] (in which the EER for 30 s, 10 s and 3 s test utterances is 1.98%, 3.47% and 9.71%).

The paper is organized as follows. How to generate the DBF from a DNN is first briefly introduced, including the two main categories, generative pre-training and discriminative fine-tuning. Then, our proposed LID systems is presented in detail. Finally, the experimental setup and results are presented and analyzed, followed by the conclusion and future work.

## Methods

### Deep Bottleneck Features

**Figure 1:**
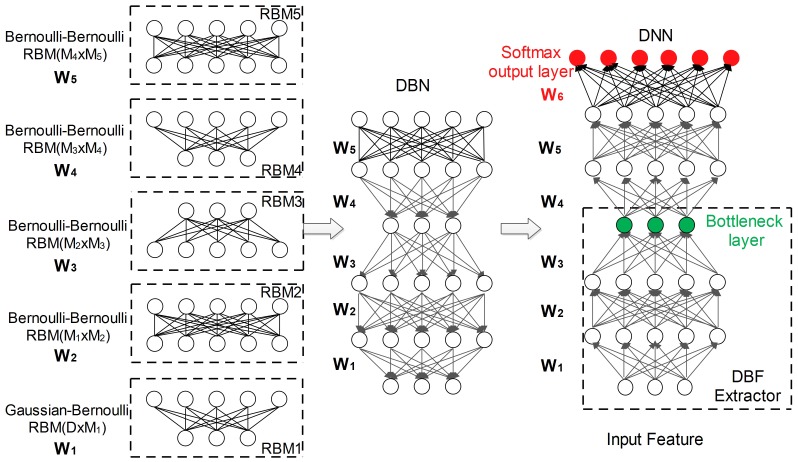
An illustration of the DNN training and DBF extraction procedure. Left: Pre-training of a stack of RBMs with the first layer hosting a Gaussian-Bernoulli RBM and all other layers being Bernoulli-Bernoulli RBMs. The inputs to each RBM are from the outputs of the lower layer RBM. Middle: The generative model DBN constructed from a stack of RBMs. Right: The corresponding DNN and DBF extractor. The DNN is created by adding a randomly initialized softmax output layer on top of the DBN, and the parameters of DNN are obtained in a fine-tuning phase. The final DBF extractor in the bottom right dashed rectangle is obtained by removing the layers above the bottleneck layer.

In this section, we discuss the DBF extraction procedure and structure as shown in Figure 1, used as an acoustic frontend for the spoken LID task. We first describe the DNN training process, including generative pre-training and discriminative fine-tuning phases, followed by the DBF extraction process. We then detail the configuration of DBF extraction for LID. Finally, we discuss the relation to several existing frame level features, e.g. SDC and PLLR.

### DNN Training

The DNN training process includes pre-training and fine-tuning phases [34]. During the pre-training phase, a generative Deep Belief Net (DBN) with stacked Restricted Boltzmann Machines (RBM) is trained in an unsupervised way. During the discriminative fine-tuning phase, a randomly initialized softmax layer is added on top of the DBN, and all the parameters are fine-tuned jointly using back-propagation (BP). Generally, the pre-training phase provides a region of the weight space that allows the fine-tuning phase to converge to a better local optimum, and reduce overfitting [35].

#### Pre-Training Phase

The basic idea of pre-training is to fit a generative DBN model to the input data. Conceptually, the DBN can be trained greedily in a layer-by-layer manner, by treating each pair of layers as a RBM [36], as shown in the left part of Figure 1. An RBM is a bipartite graph model in which the visible stochastic units are only connected to the hidden stochastic units [37].

The RBM is a two-layer structure with 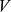 visible stochastic units 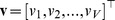, and 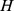 hidden stochastic units 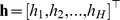. The most frequently used RBMs are the Gaussian-Bernoulli RBM and Bernoulli-Bernoulli RBM. In Bernoulli-Bernoulli RBM, 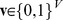 and 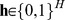 are assumed to be binary, the energy function of the state 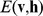 is defined as:
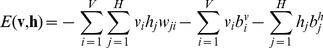
(1)


where 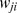 represents the weight between visible unit 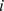 and hidden unit 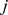, 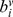 and 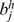 denote the real-valued biases of visible unit 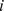 and hidden unit 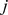 respectively. The Bernoulli-Bernoulli RBM model parameters can be defined as 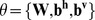, where 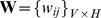, 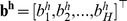 and 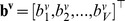. For a Gaussian-Bernoulli RBM, the visible units are real-valued which means 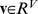, and 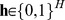 are binary. Thus, the energy function is defined as follows:
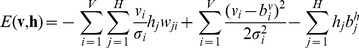
(2)


where 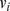 is a real-valued activity of visible unit 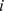. Each visible unit adds a parabolic offset to the energy function which is governed by 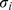. The Gaussian-Bernoulli RBM model parameter set can be defined as 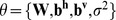 similarly, where the variance parameters 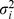 are commonly fixed to a pre-determined value instead of being learnt.

According to the energy function 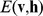 in Eq. (1)&(2), the joint probability associated with configuration 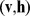 is defined as follows:
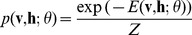
(3)


where 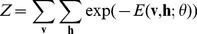
(4)


is a partition function. Given a training set, the RBM model parameters 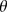 can be estimated by maximum likelihood learning via the contrastive divergence (CD) algorithm [38]. After the RBM of a lower layer is trained, the inferred states of the hidden units can be used as the visible data for training the RBM of a higher layer. This process is repeated to produce multiple layers of RBMs. Finally, the RBMs can be stacked to produce the DBN, as shown in the middle part of Figure 1.

#### Fine-Tuning Phase

The fine-tuning phase is shown in the right part of Figure 1, in which an output labelling layer is added on top of the pre-trained DBN. For a multiclass classification problem, there are 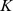 units in the output layers. In our work, these units correspond to the language-specific phonemes. Each unit corresponds to the label of input features, which converts a number of Bernoulli distributed units 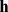 into a multinomial distribution through the following softmax function, 
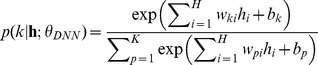
(5)


where 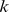 is an index over all classes, 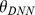 are the DNN model parameters, 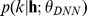 denotes the probability that the input is classified into the 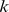-th class.

The cost function C defines the cross-entropy error between the true class label 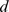 and the predicted label from the softmax operation; 
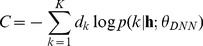
(6)


where 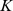 is the total number of classes, and 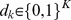 are the target variables indicating the class label with a 1-of-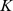 coding scheme. The BP algorithm is used to jointly tune all model parameters by minimizing the cross entropy function in Eq. (6).

### DBF Extraction

Given a trained DNN, each hidden layer proposes an internal representation of the input features. These layers can be further used to predict the phonemes or phoneme states. The DBF extractor removes the layers above the bottleneck layer, shown by the bottom right dashed rectangle in Figure 1. The advantage of a bottleneck layer is that, being smaller, it reduces the redundancy of input features and effectively reflects the relevant class label information [39]–[41].

The corresponding DBF is a vector 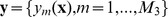, where 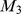 denotes the number of hidden units in the 3-rd hidden layer and 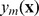 can be extracted using 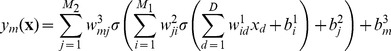
(7)


where 
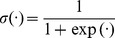
 represents the logistic sigmoid function. 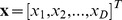 is the 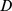 -dimensional input feature, concatenated from multiple frames of MFCC and prosodic features. 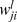 is the weight on a connection to unit 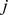 in the 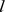-th hidden layer from unit 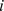 in the layer below. 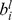 is the bias of unit 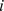 in the 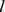-th hidden layer.

### DNN Training Settings

#### Corpus

Two separate DNNs, used for forming DBF extractors, are evaluated in this paper. The Mandarin DNN (MA-DNN) is trained from conversational telephone speech, consisting of more than 1,600,000 utterances of about 1,000 hours duration, recorded from 32,950 Mandarin speakers. The English DNN (EN-DNN) uses the well-known Switchboard corpus, consisting of the Switchboard-I training set and 20-hour Call Home English data, having about 300-hours duration.

This data will only be used to train and construct two DBF feature extractors (MA-DBF and EN-DBF). Each feature extractor will later be evaluated for LID, using completely different multilingual training and test data.

#### DNN Configuration

The DNN configuration is similar to that used for ASR [29], [41], [42]. Specifically, the feature dimension of each frame is 43, consisting of 39-dimensional MFCC+Δ MFCC+ΔΔ MFCC, and 4-dimensional pitch features corresponding to the static pitch, 1st and 2nd derivatives and voiced speech confidence respectively. The frame feature is pre-processed with Cepstral Mean Variance Normalization (CMVN). The detailed DNN structure has 1 input layer, 5 hidden layers and 1 output layer, configured as 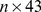 -2048 -2048 - 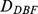 -2048 -2048 - 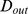. The input feature is constructed in a frame by frame manner. For each fame, the corresponding DNN input is a concatenation of the current frame with the preceding and following 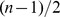 neighbouring frames. For example, if we set 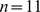, the input comprises 5 neighbouring frames before and after the center frame. 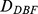 is the number of units in the bottleneck layer, which is empirically set to 43 as mentioned above. 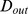 is the number of units in the output layer. In practice, 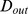 is set to 6004 and 9004 according to tri-phone tied states of Mandarin and English separately [41]. This configuration is the baseline for training the DBF extractor.

The training process is similar as that used in speech recognition [41]. During pre-training, we use 6 full sweeps through all training data for the Gaussian-Bernoulli RBM and 5 full sweeps for 4 other Bernoulli-Bernoulli RBMs. Each RBM training is implemented using CD learning with 1-step Gibbs sampling. In the fine-tuning step, we set the learning rate to a small value, i.e. 0.002, for all layers. In the fine-tuning phase, the parameters of all layers are jointly tuned using the BP algorithm according to tied-state labels obtained by a forced-alignment process using pre-trained GMM-HMMs. The fine-tuning process is iteratively executed using the following settings: 10 epochs are used for BP fine-tuning. The learning rate is fixed for the first 3 epochs, then halve for the remaining epochs. It is worthwhile to emphasize the difference between ASR and LID tasks, so we experimented extensively with different DNN configuration to find the optimal configuration of DBF extractor for performing LID.

### Relation to Existing Features

#### Relation to SDC

SDC, one of the most common acoustic features for spoken LID, is considered an extension of MFCC and PLP, which aims to capture phonemic information over a longer time-span. This extension is achieved by a simple linear transformation of several concatenated delta cepstral blocks. It is a matter of trial and error to set optimal SDC parameters, and these may vary with different LID tasks [14]. In addition, SDC is generally prone to distortion by language independent nuisance, such as speaker and channel variabilities, and specific content for a given utterance.

Similar to SDC, the DBF extractor takes the features extracted from concatenated frames as input. However, DBF exploits long-term temporal information in input features through a non-linear transformation. Futhermore, by taking into consideration the labeling information contained in the training corpus, the DBF is extracted with discriminative training, which is more robust to language-independent nuisance. Finally, DBF can be considered as a fusion of the middle-level representation between the high-level phonetic and low-level acoustic features.

### Proposed LID Systems Using DBF

In this section, we present two TV based acoustic systems to evaluate the effectiveness of the DBF for spoken LID, termed DBF-TV and PDBF-TV. The TV approach was first introduced in the context of speaker verification [24] and has become the state-of-the-art modeling technique both in speaker and language communities [25].

### DBF-TV

**Figure 2:**
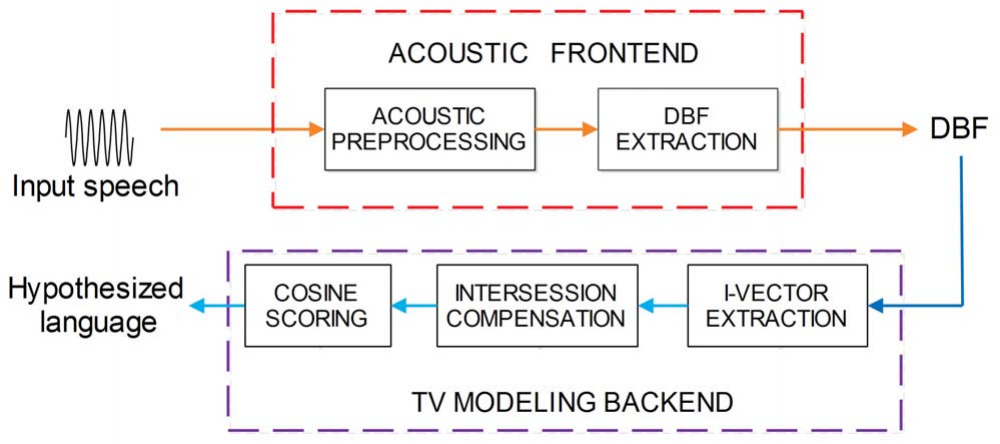
Block diagram of our proposed DBF-TV LID system. This system consists of two main phases, the acoustic frontend and TV modeling back-end.

The basic DBF-TV framework is derived from our previous work [31], and consists of two main parts, the acoustic frontend and TV modeling back-end, as shown in Figure 2. The acoustic frontend mainly consists of acoustic preprocessing and DBF extraction, as illustrated in the previous section, which transforms the multiple frames of MFCC and prosodic features into DBFs. The TV modeling back-end consists of the following phases, i-vector extraction, intersession compensation, and cosine scoring, which are described in the following paragraphs.

#### I-Vector Extraction

I-vectors are extracted via TV modeling approach, which is motivated by the success of Joint Factor Analysis (JFA) for speaker recognition task [43]. The classical JFA technique models both speaker and channel subspaces separately. However, the channel and speaker informations are difficult to separate [44]. To address this issue, TV approach was proposed to cover the total variability in an utterance using only one subspace [24]. Specifically, given an utterance, the GMM super-vector 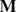, which is created by stacking the mean vectors of a GMM adapted to that utterance, can be modeled as follows 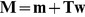
(8)


where 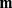 is the UBM super-vector, 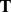 is a low rank rectangular matrix. 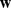 is the required low-dimensional i-vector with normal distribution 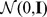.

The training process of loading matrix 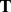 is similar to the eigenvoice method [45]. The difference is that in TV modeling, the loading matrix 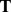 is estimated based on the variance information derived from all utterances.

#### Intersession Compensation

After i-vector extraction, two intersession compensation techniques are applied to remove the nuisance in i-vectors. The first is linear discriminant analysis (LDA) which is a popular dimension reduction method in the machine learning community. Generally, LDA is based on the discriminative criterion that attempts to define new axes minimizing the within-class variance, while maximizing the between-class variance. The LDA projection matrix 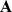 contains the eigenvectors with respect to the decreasing order of corresponding eigenvalues in decomposition. This is obtained by solving the following generalized eigenvalue problem 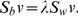
(9)


where 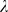 is the diagonal matrix of eigenvalues. The matrices 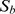 and 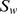 denote the between-class variance and within-class variance, respectively.
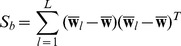
(10)

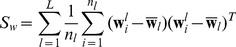
(11)


where 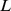 is the number of target languages, 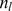 is the number of utterances for each language 
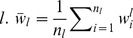
. is the mean of i-vectors for each language and 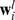 represents the 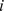-th sample of language 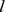.

The second intersession compensation technique we used is within-class covariance normalization (WCCN), which normalizes the cosine kernel between utterances with an inverse of the within-class covariance [24]. The within class covariance matrix 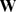 is estimated as follows: 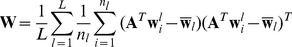
(12)


where 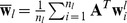 is the mean of the LDA projected i-vectors for each language. The projection matrix 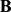 is obtained through Cholesky decomposition of matrix 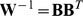. With the matrix 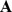 and 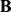, the compensated i-vector 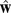 can be obtained as
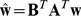
(13)


#### Cosine Scoring

After obtaining intersession compensated i-vectors, the representation of 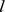-th target language 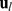 can be simply obtained by taking the mean of the corresponding i-vectors.
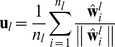
(14)


Given a test utterances, the detection score for a target language 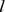 can be estimated using the cosine similarity measure between the target i-vector 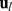 and the test i-vector 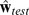: 
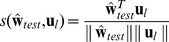
(15)


### PDBF-TV

As aforementioned, the DBF extractor is a part of the specially structured DNN, which is trained on the corpus with phonemes or phoneme states information. This labeling information may not be sufficient to cover all LID corpus due to the limited phoneme set for a special language. To address this, we propose a PDBF-TV system to further improve the LID performance.

The concept of PDBF-TV is similar to PPRLM, which aims to take advantage of complementary acoustic models. Two different PDBF-TV systems based on having different DBF extractors as parallel acoustic front ends, are proposed using two different fusion schemes: early fusion and late fusion. The early scheme conducts fusion at feature-level, where the feature from both DBF-TV systems are combined before classification. The late fusion scheme acts at a decision-level, where the outputs of the mono DBF-TV systems are integrated by the use of an averaging criteria.

**Figure 3:**
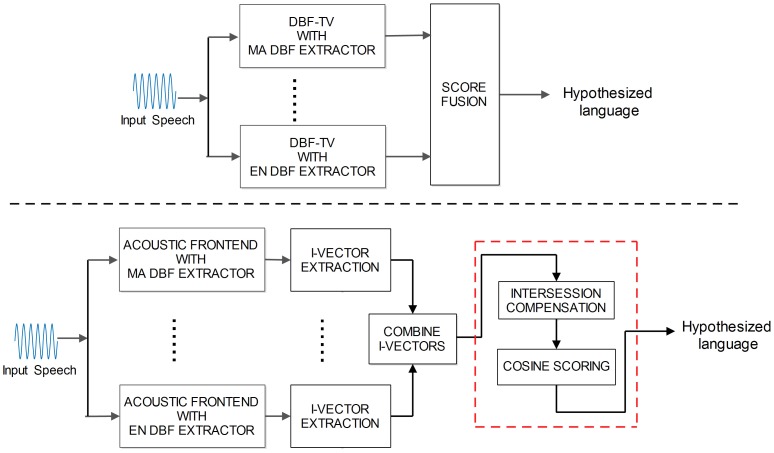
Block diagrams of two PDBF-TV LID systems. The diagram above the dashed line is PDBF-TV with later fusion. The diagram below the dashed line is the PDBF-TV with early fusion.

As shown in Figure 3, in the early fusion scheme, the features (i.e. i-vectors from different DBFs) are concatenated as the input to the TV-modeling backend. After concatenation, the following process is used in the same way as in DBF-TV, including intersession compensation and cosine scoring. In the late fusion scheme, the similarities estimated from different DBF-TV systems are averaged to form the final decision.

## Results and Discussion

### Experimental Setup

#### LID Database

To evaluate the effectiveness of the proposed DBF-based systems, we conducted extensive experiments using the LRE09 dataset, comprising 23 target languages, i.e. Amharic, Bosnian, Cantonese, Creole, Croatian, Dari, English-American, English-Indian, Farsi, French, Georgian, Hausa, Hindi, Korean, Mandarin, Pashto, Portuguese, Russian, Spanish, Turkish, Ukrainian, Urdu and Vietnamese. The training utterances for each language came from two different channels, i.e. the dataset of Conversational Telephone Speech (CTS) and narrow band Voice of America (VOA) radio broadcasts.

- CTS partition: Data from the previous evaluations conducted by NIST, including LRE 1996, LRE 2003, LRE 2005 and LRE 2007. These utterance are mainly collected from CallFriend, CallHome and Mixer databases
- VOA partition: Most of the utterances are from the NIST-provided datasets: VOA2 and VOA3.

It should be noted that the training data for each language is imbalanced. Languages such as English and Mandarin enjoy more than 100 hours of data while languages such as English-Indian are represented by less than 5 hours of data. In addition, some language data is collected from only one channel source. In implementation, we limit the training data set to at most 15 hours for each target language and divide the LID corpus into two parts: a training dataset and a development dataset. For each target language, around 80 audited segments of approximately 30 s duration are used as the development dataset, the rest are used as training.

The test utterances are also divided into three duration groups, i.e. 30 s, 10 s and 3 s, comprising 10,376, 10,427 and 10,375 speech utterances respectively.

The LRE09 dataset is very challenging in that 1) There are 23 languages, far more than in the previous evaluations. 2) Some language pairs are highly confused, such as Hindi and Urdu, Russian and Ukrainian. 3) The data is collected from different channel sources, and is highly imbalanced.

#### Performance Measurement

The core test of LRE09 is the language detection task: Given a segment of speech and a hypothesized target language, determine whether the target language is spoken in the test segment or not [9]. According to the duration of the test utterance, the performance is evaluated on 30 s, 10 s and 3 s of data respectively.

Three different metrics are used to assess the performance of LID, all evaluating the capabilities of one-versus-all language detection. The first metric is the average decision cost function (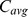) [9], which is a measure of the cost of taking bad decisions. The second one is the DET curves [46], which are used to represent the range of possible system operating points of detection systems and measure the system discrimination capability. We also compute the classical equal error rate (EER) as the performance measure.

#### LID Systems

The LID systems used for comparison are SDC-TV and PPR-LM, which rely on conventional acoustic and phonotactic features respectively.

In the SDC-TV baseline system, the SDC are extracted as follows: 1) MFCC features are extracted for each 20 ms analysis frame, with 10 ms frame shift. 2) The SDC features comprise the static and stacked MFCCs with parameter 7-1-3-7 [15]. 3) The non-speech frames are gated out by using voice activity detection (VAD). 4) SDC features are normalized to a standard distribution. The TV space is estimated using a GMM-UBM with 2048 Gaussian components and with the dimension of the i-vector set to 400 [25].

The PPRLM baseline system is implemented as described in Xu et.al. [27], with different PR frontends, i.e. BUT TRAPs/NN phone decoders for Hungarian (HU) and Russian (RU) [47].

Using the proposed DBF extractor for front end feature vector formation, we implemented the two DBF-based acoustic systems, i.e. DBF-TV and PDBF-TV. Furthermore, we built a phonotactic representation using the GMM-HMM based PR, trained using the DBF which will be compared against published PPRLM systems.

These systems will now be evaluated and compared in the following section.

### Comparison with Baseline

The proposed MA DBF-TV and EN DBF-TV systems (i.e. with DBF extractors tuned on Mandarin and English speech respectively) are now compared with the baseline SDC-TV system. The DBF extractor in each DBF-TV system is configured to be 5-1-5 for inputs, which consists of 11 frames of 43-dimension MFCC and prosodic features, and 43 hidden nodes for output. In addition, we also compare against the MIT SDC-TV setup having state-of-the-art performance. The performance published in [25] was tested on exactly the same evaluation data set. Results are shown in Table 1, where it is evident that our SDC-TV implementation is comparable to the MIT SDC-TV system. This implies that, since they having the same acoustic frontend (i.e. SDC), their back-end TV modelling implementations are also similar.

**Table 1:** Comparison of Performances between DBF-TV system and SDC-TV system on LRE09.

	30 s		10 s		3 s	
system	EER	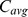	EER	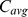	EER	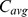
SDC-TV	2.08	2.07	5.35	5.32	16.74	16.70
MIT SDC-TV [25]	2.40	N/A	4.80	N/A	14.20	N/A
MA DBF-TV	1.51	1.37	2.62	2.59	9.28	9.18
EN DBF-TV	1.42	1.41	2.67	2.61	10.14	10.04

Most importantly, we can see clearly in Table 1 that the performances of the DBF-TV systems is very promising. For the MA DBF-TV system, the EERs of 30 s, 10 s and 3 s test utterances are 1.51%, 2.62% and 9.28% respectively, whereas for the EN DBF-TV system, they are 1.42%, 2.67% and 10.14%. The relative improvements of DBF-TV over the baseline range from 62.7% to 82.7%, with the highest improvements seen for 10 s test utterances.

**Figure 4:**
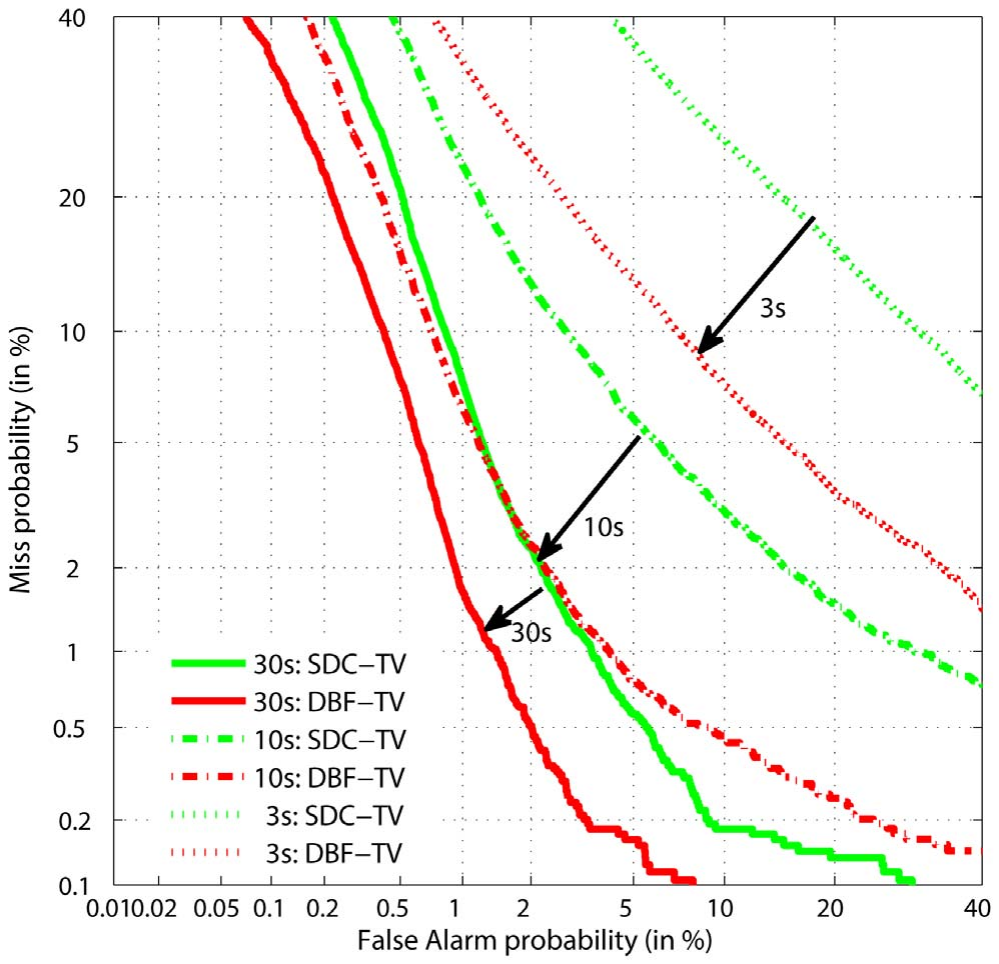
DET curves comparison between MA DBF-TV and SDC-TV.

Since we have established that the back-end TV modelling is similar in each case, this significant performance improvement is mainly due to ability of the DBF frontends. It demonstrates that the DBF features are powerful and have good discriminative and descriptive capabilities for the LID. To explore further, Figure 4 shows a DET curve comparison between the SDC-TV and MA DBF-TV systems.

In the DBF-TV systems, the configuration of the DBF extractor is fixed. Despite the significant performance improvement seen, this configuration may not be optimal. In the following subsection, we therefore compare the performance of different DBF extractor configurations, and propose an optimal configuration for the LRE09 dataset.

### DBF Configurations

In this section we construct experiments to evaluate the effect of DBF extractor configurations, using the MA DBF-TV system as baseline. The experiments separately assess different input temporal window sizes as well as the number of hidden nodes for the DBF extractor output, in order to find an optimal configuration for the LRE09 dataset.

#### Temporal Window Size Investigation

It is known that temporal context information plays an important role for LID performance. For SDC, extensive trials have been conducted [14], leading to a relatively stable and optimal configuration. Taking a similar approach, we experimentally assess the performance of different temporal window size configurations for DBF extraction. The resulting LRE09 performance is evaluated for four different DBF extractor configurations, i.e. 5-1-5, 10-1-10, 15-1-15 and 20-1-20, and shown in Table 2 with best results shown in bold text. We can see that, for 30 s and 10 s test utterances, the 10-1-10 DBF extractor configuration (i.e. a temporal window size of 21) performs best whereas for 3 s test utterances, the 5-1-5 DBF extractor configuration performs slightly better. Taken overall, the 10-1-10 configuration with window size 21 is optimal. In fact, this result coincides with the configuration of conventional SDC, i.e. 7-1-3-7 with window size 21.

**Table 2:** Comparison of Performances between different temporal context sizes using 43-dimensional DBF on LRE09.

	30 s		10 s		3 s	
Temporal Window Size	EER	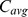	EER	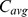	EER	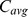
5-1-5	1.51	1.37	2.62	2.59	9.28	9.18
10-1-10	1.31	1.22	2.36	2.34	9.64	9.60
15-1-15	1.39	1.29	2.47	2.43	9.72	9.69
20-1-20	1.34	1.23	2.49	2.44	10.03	10.00

#### DBF Extractor Output Hidden Nodes Investigation

**Figure 5:**
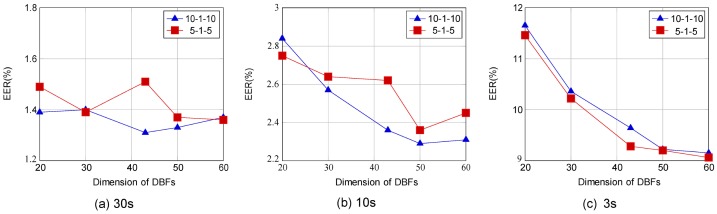
EER obtained from the MA DBF-TV system based on different dimensions of DBF on LRE09. Left panel shows the results of 30 s. Middle panel shows the results of 10s. Right panel shows the results of 3 s.

In order to assess the effect of the number of hidden nodes at the output of the DBF extractor, we construct several experiments. Two baseline DBF extractor configurations are used, having 10-1-10 and 5-1-5 temporal input windows respectively (since these yielded best performance for the 30 s, 10 s, and 3 s test utterances in the previous subsection). The EER of 30 s, 10 s and 3 s test utterances are determined for each for hidden node numbers ranging from 20 to 60 (with 43 being the nominal value, set to match the dimension of the input vector). The results are plotted in Figure 5. We can conclude that, for 30 s utterances, the number of hidden nodes in the test does not directly affect LID performance. For 10 s and 3 s test utterances, performance tends to improve as the number of hidden nodes increases. Performance improvement in those cases appears to saturate around dimension 50. Therefore an optimal configuration is chosen: an input of 10-1-10 with temporal window size 21, and 50 hidden nodes in the DBF output layer. This configuration can achieve an EER performance of 1.33%, 2.29% and 9.22% on 30 s, 10 s, 3 s test utterances respectively.

With longer test utterances, the statistics of speech content may already be sufficient for LID. However for shorter utterances, with insufficient statistics, the additional ability of the DBF extractor appears to be more effective at improving system performance.

As a summary, our study on the input and output of DBF extractor is consistent with previous studies, such as the configuration of SDC. And with powerful modelling capability of DNN, the system performance can be significantly improved with optimal configuration.

### Performance of the Proposed PDBF-TV System

This section presents the results of the proposed PDBF-TV system which combines both the MA and EN DBF extractors in parallel. Both use the optimal configuration obtained in the experiments of the previous subsections. Two schemes are used for fusion, one is early-fusion where the i-vectors are concatenated for the final LID feature vector, and the other is later-fusion which performs a weighted mean of the output scores. Results from these two schemes are given in Table 3, with best scores for each test given in bold text. From this, we can see that both early fusion and later fusion schemes achieve an improvement over the baseline DBF-TV system, however early fusion performs slightly better – although at the cost of a slightly increased computational complexity.

**Table 3:** Comparison of Performance between two different PDBF-TV systems on LRE09.

	30 s		10 s		3 s	
System	EER	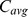	EER	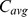	EER	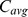
MA DBF-TV	1.33	1.25	2.29	2.27	9.22	9.17
EN DBF-TV	1.38	1.27	2.58	2.56	9.98	9.91
PDBF-TV1 (later)	1.31	1.28	2.24	2.20	7.45	7.45
PDBF-TV2 (early)	1.22	1.16	2.09	2.05	7.93	7.87

### Performance Comparison with State-of-the-Art

To further demonstrate the effectiveness of the proposed DBF, we now investigate fusing the acoustic and phonotactic approaches. The acoustic approach is the PDBF-TV2 system as defined in the previous subsection. The phonotactic representation is constructed using 4 PRs, i.e. RU, HU, MA and EN.

**Figure 6:**
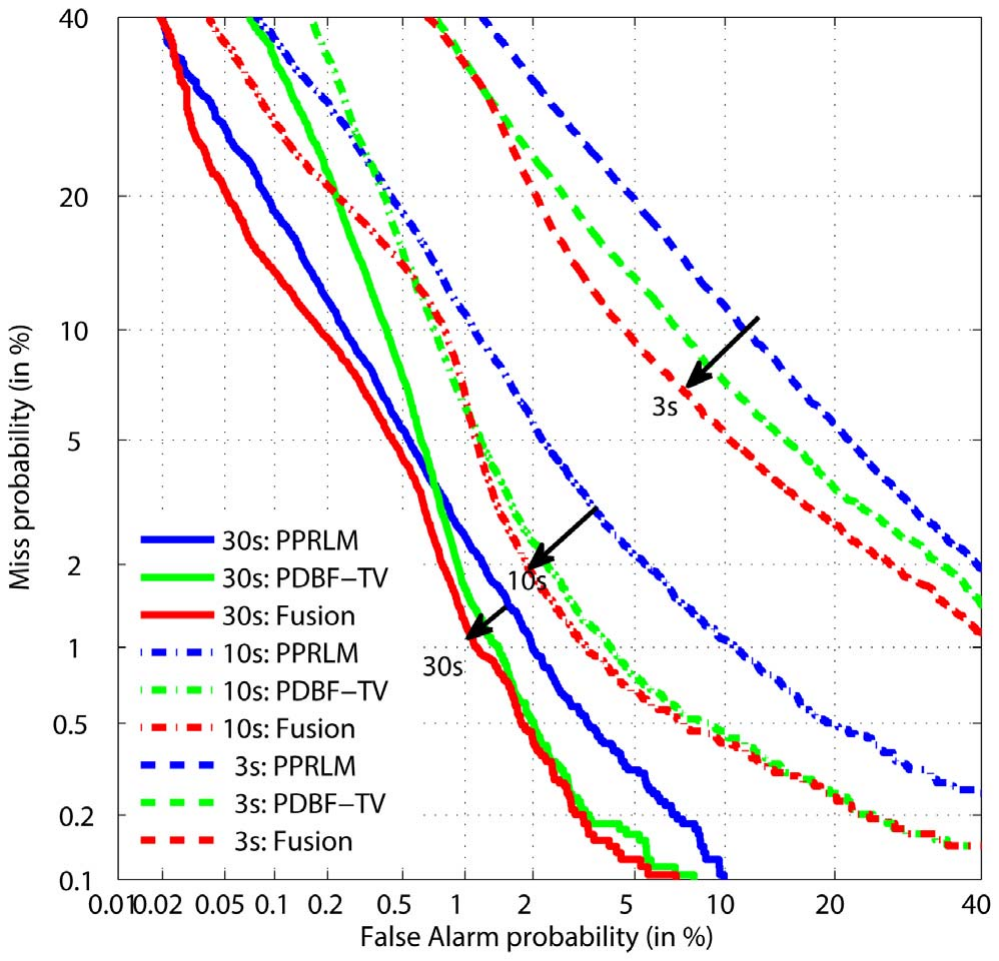
DET curves comparison between PPRLM, PDBF-TV (MA+EN) and their fusion on LRE09.

The RU and HU phone recognizers are from Brno University of Technology (BUT), trained using TRAP features and a NN method [47]. The MA and EN recognizers are trained with the corresponding DBFs using classical GMM-HMM training. The experimental results are shown in Table 4, with best scores shown in bold text. From this we can see that the performance of DBF/GMM-HMM based PRLM, P3 and P4, is comparable to the TRAPs/NN based PRLM, P1 and P2. The performance of both F1 and F2 PPRLM systems is inferior to the DBF-TV and PDBF-TV systems. By fusing the outputs of all these acoustic and phonotactic systems, EERs of 1.08%, 1.89% and 7.01% can be achieved. We also list the results from the MITLL [26] and BUT-AGNITIO [48] systems, both of which similarly fuse acoustic and phonotactic methods. It is evident that the fusion results from the proposed system significantly exceed the performance of these reported state-of-the-art LID systems, especially for short duration test utterances. In Figure 6, DET plots of the PPRLM, PDBF-TV2 and fusion systems are shown, again highlighting the effectiveness of the proposed DBF.

**Table 4:** Fusion results between PDBF-TV system with PPRLM system on LRE09.

	30 s		10 s		3 s	
System	EER	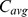	EER	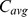	EER	
P1: PRLM with RU	2.42	2.40	6.42	6.38	18.92	18.70
P2: PRLM with HU	2.62	2.62	6.65	6.62	18.88	18.82
F1: PPRLM(P1+P2)	1.78	1.78	4.70	4.65	15.24	15.15
P3: PRLM with MA	3.08	3.03	7.79	7.78	21.93	21.65
P4: PRLM with EN	2.58	2.58	6.09	6.07	17.30	17.29
F2: PPRLM(P3+P4)	2.13	2.10	4.51	4.46	13.50	13.45
F3: PPRLM(F1+F2)	1.53	1.49	3.31	3.29	10.71	10.65
F4: PDBF-TV2	1.22	1.16	2.09	2.05	7.93	7.87
Fusion:(F3+F4)	1.08	1.05	1.89	1.85	7.01	6.96
MITLL LRE09 [26]	N/A	1.64	N/A	3.14	N/A	10.50
BUT-AGNITIO LRE09 [48]	N/A	1.57	N/A	2.76	N/A	10.22

## Conclusions

In this paper, we have proposed and evaluated the use of DBF for spoken LID. The DBF extractor is generated from a structured DNN having a narrow internal bottleneck layer. It has been shown that DBFs can form a low-dimensional compact representation of the original inputs, and have a powerful descriptive and discriminative capability, when the DNN is carefully constructed and trained. Two acoustic approaches, i.e. DBF-TV and PDBF-TV, were constructed and evaluated to demonstrate the effectiveness of the proposed DBF. Compared to conventional SDC-TV approaches, the experimental results on the challenging LRE09 core test show significant performance improvement, especially for short duration utterances. Furthermore, different configurations of DBF extractor have been studied, with an optimal system being proposed for spoken LID. By fusing the output of phonotactic and acoustic representations based on DBFs, final results are achieved which outperform existing published state-of-the-art systems.

It is believed that this work is the first step towards effective representations for LID through applying the ideas of deep learning. In future, several extensions may be worthwhile. Firstly, all experiments in this paper are carried out on the LRE09 closed-set task. It is worth examining the effectiveness of DBF on even more challenging LID tasks, such as dialect recognition, and open-set tasks. Secondly, there are many parameters in the DNN structure that are empirically determined. The work presented in this paper focuses on the input and output parameters of the corresponding DBF extractor, yet it may be interesting to further investigate other configuration options effective for spoken LID, such as the number of nodes in hidden layers as well as the number of hidden layers. Thirdly, this work mainly considers acoustic approaches. For the phonotactic approach, only PPRLM systems based on DBF were evaluated. Further performance improvement may be achievable by using more powerful modelling techniques, such as SVM and Binary Tree.
